# The Role of Viscosity and Fermentability of Dietary Fibers on Satiety- and Adiposity-Related Hormones in Rats

**DOI:** 10.3390/nu5062093

**Published:** 2013-06-07

**Authors:** Natalia Schroeder, Len F. Marquart, Daniel D. Gallaher

**Affiliations:** Department of Food Science and Nutrition, University of Minnesota, St. Paul, MN 55108, USA; E-Mails: schr0620@umn.edu (N.S.); lmarquar@umn.edu (L.F.M.)

**Keywords:** dietary fiber, viscosity, fermentation, GLP-1, ghrelin, PYY, leptin, insulin, satiety

## Abstract

Dietary fiber may contribute to satiety. This study examined the effect of two dietary fiber characteristics, small intestinal contents viscosity and large intestinal fermentability, on satiety-and adiposity-related hormones in rats. Diets contained fiber sources that were non-viscous, somewhat viscous, or highly viscous, and either highly fermentable or non-fermentable, in a 2 × 3 factorial design. In the fed state (2 h postprandial), rats fed non-fermentable fibers had significantly greater plasma GLP-1 concentration than fermentable fibers. In the fasted state, among non-fermentable fibers, viscosity had no effect on GLP-1 concentration. However, among fermentable fibers, greater viscosity reduced GLP-1 concentration. Plasma peptide tyrosine tyrosine (PYY) concentrations in the fasted state were not influenced by the fermentability of the fiber overall, however animals consuming a fructooligosaccharide greater PYY concentration. In both the fed and fasted states, rats fed non-fermentable fibers had a significantly lower plasma ghrelin concentration than rats fed fermentable fibers. In the fasted state, rats fed non-fermentable fibers had a significantly lower plasma leptin concentration than rats fed fermentable fibers. Thus, fermentability and viscosity of dietary fiber interacted in complex ways to influence satiety- and adiposity-related plasma hormone concentrations. However, the results suggest that highly viscous, non-fermentable fibers may limit weight gain and reduce adiposity and non-fermentable fibers, regardless of viscosity, may promote meal termination.

## 1. Introduction

The prevalence of obesity in the U.S. remains at high levels, at approximately 36% as of 2009–2010 [1]. In spite of considerable effort, attempts to reduce this high prevalence of obesity have not yet been effective. It is likely that a multifactorial approach will be necessary to impact this high prevalence. Modulation of food intake by consuming foods of high satiety value may be one approach to help reduce obesity, as this has been shown to cause earlier termination of a meal or to reduce intermeal food intake [2].

One food component shown to have satiating or satiety effects is dietary fiber. A high fiber intake has been reported to increase the perception of satiation [3], enhance subjective satiety [4], delay the onset of hunger [5], and decrease energy intake [6]. Additionally, those who have greater intakes of fiber tended to weigh less or experience less weight gain over time [6,7,8,9,10]. However, some have found fiber to have no effect on subjective satiety [11,12,13,14]. The different types and amounts of fibers used in these studies may contribute to the inconsistent results, as different types of dietary fibers may not influence satiety and food intake equally.

Although dietary fiber is a complex mixture of indigestible plant materials, most of the physiological benefits from fiber are attributed to two characteristics of dietary fiber, viscosity in the small intestine and fermentability in the large intestine. Viscous fibers create gastric distention and delay gastric emptying [15], leading to a sense of fullness due to greater volume within the stomach [16]. Food products containing dietary fiber with a high viscosity, such as psyllium [17], carrageenan [18], β-glucans [19] or a modified cellulose [20] have shown satiating effects. Rats [21] and humans [17] fed a high viscosity pre-meal were found to consume less food at a subsequent test meal than when they consumed a low viscosity meal. A recent study found subjects had greater ad libitum intake after consuming a low viscosity *versus* a high viscosity food product [18]. However, viscous fibers naturally present in food, such as β-glucans and pectins, are also highly fermentable by the intestinal microflora in the large intestine, leading to formation of short chain fatty acids (SCFA). SCFA have been hypothesized to promote satiety [22], although a recent review concludes that this concept is not supported [23]. Thus, with naturally occurring viscous fibers, it is uncertain whether effects on satiety would be due to its viscosity or its fermentability.

Satiation is defined as the feeling of fullness during the consumption of a meal, which leads to meal termination. Satiety is commonly defined as the feeling of fullness from the consumption of a previous meal, which inhibits eating between meals [2]. Therefore, the consumption of satiating foods and/or foods with a high satiety value may help to control food intake by causing earlier termination of a meal or reducing intermeal food intake [2]. A number of hormones have now been identified that signal satiation or satiety [24]. Glucagon-like peptide (GLP)-1, located in the L cells of the ileum and proximal colon, enhances satiety and reduces food intake when administered to normal subjects [25]. Similarly, peptide tyrosine tyrosine (PYY), secreted from the same L cells as GLP-1, also reduces food intake as well as delaying gastric emptying [26]. Ghrelin is unique among the gut hormones in that it exerts an orexigenic effect, thereby signaling hunger and stimulating food intake [27]. Dietary fiber has the potential to influence satiation and satiety by changing the secretion of these satiety-related hormones as well as the adipokine leptin [19,28,29]. Animal studies feeding fermentable fibers such as inulin, lactitol, resistant starch, and fructooligosaccharides have reported less weight gain, increased concentration of plasma GLP-1, proximal colon GLP-1 and plasma PYY [28,29,30,31,32,33,34,35], in addition to decreased concentrations of serum ghrelin [29] and circulating leptin levels [36].

Studies examining fiber and satiety in terms of either the viscosity or the fermentability of fiber demonstrate that these characteristics do influence satiety compared to non-viscous and/or non-fermentable controls. However, when studies employed a viscous fiber, the fiber was often also fermentable. Likewise, if the fiber examined was fermentable, there was no clear comparison with a viscous non-fermentable fiber. Therefore, which of these two characteristics—viscosity or fermentability—most influences satiety-related hormones remains uncertain. Indeed, it may be both, albeit by different mechanisms. Therefore, the objective of this study was to examine in a systematic fashion the effect of these two dietary fiber characteristics—small intestinal contents viscosity and large intestinal fermentability—on plasma and tissue concentrations of satiety-related hormones in rats.

## 2. Experimental Section

### 2.1. Animals and Diets

**Figure 1:**
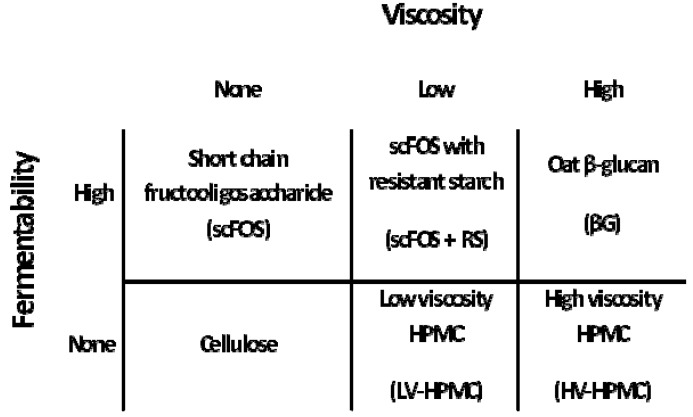
Dietary fiber types used in the experimental diets to produce a range of intestinal viscosities, with or without large intestinal fermentation. Each fiber type was present in the diets at 5%.

Male Wistar rats were housed in wire-bottom stainless steel cages in a temperature-controlled environment (12-h light/dark cycle). Animal use was approved by the Institutional Animal Care and Use Committee (study number: 0705A09168, approved 4/29/2008) and complied with the University of Minnesota Policy on Animal Care. Rats were fed diets modified from the AIN-93G purified diet [37] that varied only in the source of dietary fiber (Table 1). The fiber sources were either non-fermentable or highly fermentable, each source producing either no viscosity, low viscosity, or high viscosity within the small intestinal contents, resulting in a 2 × 3 factorial design (Figure 1). The non-fermentable fibers were cellulose (no viscosity), a low viscosity hydroxypropyl methylcellulose (LV-HPMC; a 1:3 mixture of 100LV:3LV HPMC grades, Dow Chemical Co., Midland, MI, USA), or a high viscosity HPMC (HV-HPMC; a 1:1 mixture of 4M and 15M, Dow Chemical Co., Midland, MI, USA). The fermentable fibers were short chain fructooligosaccharide (no viscosity) (scFOS; NutraFlora P-95 short-chain fructooligosaccharides, GTC Nutrition, Golden, CO, USA), scFOS with resistant starch (low viscosity) (scFOS + RS; NutraFlora P-95 with Hi-maize 260, 82.6% scFOS and 17.4% resistant starch, GTC Nutrition), or oat beta-glucan (high viscosity) (βG; OatVantage, GTC Nutrition). The diets contained 5% (w/w) experimental fiber and 3% (w/w) cellulose for a total dietary fiber content of 8% (w/w). The composition of the macronutrients in the diets was adjusted such that the concentration of all macronutrients was essentially constant among the diets.

### 2.2. Experimental Design

Rats were adapted to the cellulose diet for 3 days, then randomly assigned to one of the six diet treatments (*n =* 10–12 per group). The experimental diets were fed for 6 weeks. Animals had free access to food and water at all times. Body weights were measured weekly and food intake was determined weekly for the first 4 weeks. At week four, rats were fasted approximately 14–16 h overnight and then blood collected from the retro-orbital sinus. Blood was collected into tubes containing EDTA with an inhibitor cocktail of aprotinin, 4-(2-aminoethyl) benzenesulfonyl fluoride hydrochloride, Na_2_EDTA, and dipeptidylpeptidase IV (DPP-IV) inhibitor and centrifuged at 3000× *g* for 20 min at 4 °C. Plasma was stored at −80 °C.

**Table 1:** Diet composition.

Diet components (g/kg)	Cellulose	LV-HPMC	HV-HPMC	scFOS	scFOS + RS	βG
Fiber source	50	50	50	53.9	71	92.6
Sucrose	100	100	100	98.06	99.01	99.07
Corn starch	449.5	449.5	449.5	449.5	436.08	434.7
Casein	200	200	200	200	199.65	188.89
Cellulose	30	30	30	30	30	30
Mineral Mix	35	35	35	35	35	35
Vitamin Mix	10	10	10	10	10	10
l-cysteine	3	3	3	3	3	3
Choline Bitartrate	2.5	2.5	2.5	2.5	2.5	2.5
TBHQ	0.014	0.014	0.014	0.014	0.014	0.014
Soybean Oil	120	120	120	120	120	120
Total Weight	1000.014	1000.014	1000.014	1001.974	1006.254	1015.774
% Carbohydrate	54.95	54.95	54.95	54.84	54.61	54.10
% Protein	20.00	20.00	20.00	19.96	19.88	19.69
% Fat	12.00	12.00	12.00	11.98	11.93	11.81
% Fiber	8%	8%	8%	8%	8%	8%
Energy (kcal/g)	4.08	4.08	4.08	4.07	4.05	4.01

After two more weeks, rats were again fasted approximately 14–16 h overnight and then given a 4 g meal the next morning. Two hours after presentation of the meal, the rats were anesthetized, opened by laparotomy, organs were harvested, and blood collected by cardiac puncture and placed into tubes containing EDTA and the above-mentioned inhibitor cocktail. The cecum, cecal contents, 3 cm of the proximal colon and one epididymal fat pad were excised, flushed with distilled water, weighed, immersed in liquid nitrogen, and stored at −80 °C. The small intestine was excised and the intestinal contents collected by finger stripping.

### 2.3. Small Intestinal Contents Viscosity

The small intestinal contents were centrifuged at 20,000× *g* for 45 min at 37 °C. The contents supernatants were collected and viscosity of a 0.5 mL sample measured on a cone/plate viscometer (Brookfield Engineering Laboratories Inc., Middleboro, MA, USA) at 37 °C at all possible shear rates between 1.15 and 230 s^−^^1^ within 6–7 h of collection. Due to the non-Newtonian behavior of viscous dietary fibers (pseudoplastic flow), viscosity values *vs.* shear rate were plotted on a log-log scale. Viscosity was estimated by extrapolation of the regression line to a shear rate of 23.0 s^−^^1^.Viscosity was expressed as millipascal seconds (mPa·s).

### 2.4. Plasma and Tissue Hormone Measurements

Concentrations of plasma GLP-1 and proximal colon GLP-1 protein amount were measured using an ELISA kit specific for GLP-1 (7–36) amide (EMD Millipore, Billerica, MA, USA). Extraction of GLP-1 amide from proximal colon tissue was carried out as described by Cani *et al.* [1]. Plasma concentrations of ghrelin and leptin were measured using commercial ELISA kits (EMD Millipore). Plasma PYY was determined using a rat-specific commercial RIA kit (Linco Research).Plasma insulin was determined using a rat-specific insulin RIA (for fed state) and a sensitive rat insulin RIA (for fasted state) (EMD Millipore).

### 2.5. Statistical Analyses

Statistical differences among groups were analyzed by two-way analysis of variance to determine the effect of the fiber characteristics on the outcome variables, using viscosity (none, low, high) and fermentability (yes, no) as the factors. A one-way analysis of variance was completed to determine the effect of treatments on the outcome variables, using diet and time block as factors. A one-way analysis of variance was conducted to examine the effect of viscosity and fermentability on the outcome variables. The small intestinal contents supernatants display non-Newtonian behavior and thus were analyzed using non-parametric statistics (ranked viscosity). Pearson correlation coefficients were used to determine associations between concentrations of hormones and intestinal contents viscosity. To determine if the satiety- and adiposity-related hormone concentrations differences explain the differences in food intake, a stepwise-regression analysis was conducted to determine the association between the satiety- and adiposity-related hormones, in both fasted and fed states, and average food intake. *p* < 0.05 was considered as statistically significant. Data are presented as mean ± SEM. One animal’s values were excluded due to extreme outliers.

## 3. Results

### 3.1. Food Intake

There were no differences among diet groups for food intake at any time point (*p* > 0.05) or for average daily food intake (*p =* 0.188); however, there was a trend for a difference at week one (*p =* 0.059). There was no statistically significant main effect of either viscosity or fermentability at any time point for food intake or average daily food intake (*p* > 0.05) (Table 2).

**Table 2:** Effect of fiber viscosity and fermentability on body weight, food intake, tissue and cecum contents weight, and intestinal contents supernatant viscosity ^1^.

Para-meter ^2^	No Viscosity		Low Viscosity		High Viscosity		Significant Main Effects ^3^
	Not Fermentable	Fermentable	Not Fermentable	Fermentable	Not Fermentable	Fermentable	
	Cellulose	scFOS	LV-HPMC	scFOS + RS	HV-HPMC	βG	
Final Body Weight, g	389.2 ± 11.1 ^ac^	361.4 ± 11.0 ^bc^	370.9 ± 12.8 ^abc^	389.7 ± 10.8 ^a^	351.5 ± 6.8 ^b^	383.8 ± 9.5 ^ac^	V × F Interaction *p =* 0.017
Average Daily Food Intake, g	20.35 ± 0.21	19.64 ± 0.64	20.38 ± 0.54	21.11 ± 0.40	19.64 ± 0.34	20.26 ± 0.46	NS
Cecum Weight (empty), g	0.60 ± 0.04 ^a^	1.04 ± 0.06 ^d^	0.91 ± 0.11 ^bcd^	0.78 ± 0.05 ^bc^	0.73 ± 0.05 ^ab^	0.91 ± 0.06 ^cd^	F (*p =* 0.004); V × F Interaction *p* <0.001
Cecal Contents Weight, g	1.22 ± 0.17 ^a^	3.03 ± 0.42 ^c^	1.31 ± 0.17 ^a^	1.61 ± 0.16 ^ab^	1.89 ± 0.71 ^ab^	2.46 ± 0.32 ^bc^	F *p =* 0.005
Proximal Colon Weight, g	0.21 ± 0.01	0.18 ± 0.01	0.23 ± 0.02	0.21 ± 0.03	0.23 ± 0.02	0.19 ± 0.01	F *p =* 0.058
Epididymal Fat Pad Weight, g	3.17 ± 0.22 ^c^	2.35 ± 0.23 ^ba^	2.74 ± 0.26 ^abc^	2.94 ± 0.26 ^bc^	2.13 ± 0.22 ^a^	2.60 ± 0.22 ^abc^	V × F Interaction *p =* 0.023
Small Intestinal Contents Supernatant Viscosity, mPa·s	12 ± 3 ^a^	18 ± 5 ^a^	43 ± 7 ^b^	35 ± 13 ^b^	201 ± 125 ^c^	210 ± 65 ^c^	V *p* <0.0001

^1 ^Values are mean ± SEM, *n =* 10–12 per group; ^2 ^Numbers within a row having different letter superscripts differ significantly (*p* <0.05); ^3 ^Abbreviation: V, viscosity; F, fermentability; NS, not significant.

### 3.2. Body Weight

Initial body weight among diet groups was not significantly different (mean range 117.6 g to 119.6 g). Body weight at weeks one, two, three, four and five were not significantly different among diet groups (*p* > 0.05). However, diet groups significantly differed in final body weight (*p =* 0.029) (Table 2). Animals fed the HV-HPMC diet had the lowest final body weight whereas animals on the scFOS + RS diet had the heaviest final body weight. There was a statistically significant correlation between the group means of final body weight and the group means of average daily food intake (*r* = 0.885, *p =* 0.0192).

There were no significant main effects of viscosity or fermentability among the diet groups for initial body weight, or body weight for weeks one through four (*p* > 0.05). There was a significant viscosity × fermentation interaction for body weight at week five (*p =* 0.033) and final body weight (*p =* 0.017) (Table 2).

### 3.3. Organ and Tissue Weight

Empty cecum weight statistically differed among diet groups (*p* < 0.0001) (Table 2). Rats on the cellulose diet had a significantly lighter cecum weight than rats on all other diets except HV-HPMC. There was a significant main effect of fermentability, with fermentable fiber groups having heavier cecum weights than the non-fermentable fiber groups, and a viscosity × fermentability interaction.

Cecal contents weight differed significantly among diet groups (*p =* 0.006) (Table 2). Rats on the scFOS diet had significantly heavier cecal contents than rats on the cellulose, LV-HPMC, HV-HPMC, and scFOS + RS diets. Further, rats on the βG diet had significantly heavier cecal contents than rats on the cellulose and LV-HPMC diets. There was a significant main effect of fermentability (*p =* 0.005) for cecal contents weight, with fermentable fiber groups having significantly heavier cecal contents than the non-fermentable fiber groups, but no main effect of viscosity (*p =* 0.107).

Proximal colon weight did not significantly differ among the diet groups (*p =* 0.216) (Table 2). However, there was a strong trend for an effect of fermentability (*p =* 0.058) for colon weight, with non-fermentable fiber groups trending toward heavier colons than the fermentable groups, but no main effect of viscosity (*p =* 0.311).

Epididymal fat pad weight significantly differed among diet groups (*p =* 0.036) (Table 2).The HV-HPMC group had the lightest epididymal fat pad weight, which differed significantly from cellulose and scFOS + RS, whereas the cellulose group had the heaviest. Epididymal fat pad weight showed a significant viscosity × fermentability interaction (*p =* 0.023).

### 3.4. Small Intestinal Contents Viscosity

Small intestinal contents supernatant viscosity differed significantly among the diet groups (*p* < 0.0001) (Table 2). The small intestinal contents supernatant viscosities fell into three distinct levels, thus justifying their categorization into no, low and high viscosity groups. Contents supernatants from animals fed βG and HV-HPMC diets were the most viscous. Contents supernatants from animals fed scFOS + RS and LV-HPMC were moderately viscous whereas contents supernatants from animals fed scFOS and cellulose had almost no viscosity.

As expected, there was a significant effect of viscosity (*p* <0.0001) and no effect of fermentability (*p =* 0.583) on contents supernatant viscosity. The no, low, and high viscosity groups were significantly different from one another (*p* < 0.001).

### 3.5. GLP-1

Overall, there was no statistically significant difference in plasma GLP-1 concentrations between the fasted and fed states. Further, neither viscosity nor fermentability influenced the difference in concentration of plasma GLP-1 between the fasted and fed states.

#### 3.5.1. Fasted State

**Figure 2:**
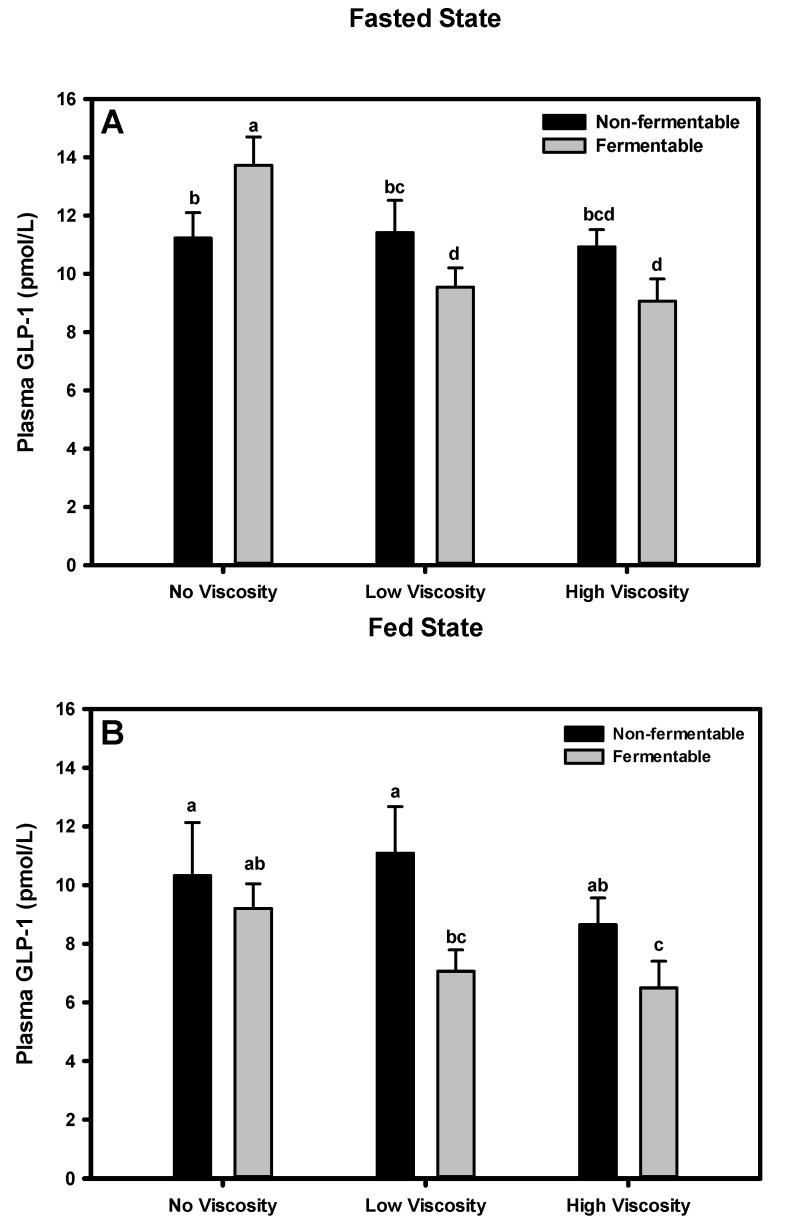
Effect of dietary fiber viscosity and fermentability on plasma GLP-1 concentrations in the fasted state (**A**) and fed state (**B**). Values are mean ± SEM, *n* = 10–12 per group. Bars having different letters differ significantly (*p* < 0.05). In the fasted state, there was a significant viscosity by fermentability interaction (*p* = 0.041). In the fed state, there was a significant main effect of fermentability (*p* = 0.021). The non-fermentable fibers, in order of increasing viscosity were cellulose, LV-HPMC, and HV-HPMC. The fermentable fibers, in order of increasing viscosity, were scFOS, scFOS + RS, and βG.

GLP-1 concentrations among diet groups differed as animals fed the scFOS diet had significantly greater GLP-1 concentrations than animals fed all other diets (Figure 2). There was a significant viscosity × fermentability interaction for plasma GLP-1 in the fasted state (*p =* 0.016).That is, the response of GLP-1 to fermentation was dependent on the viscosity. scFOS, the fermentable fiber with no viscosity, had the greatest plasma GLP-1 concentration, whereas in the diets containing fermentable fibers with some viscosity, GLP-1 concentrations were the lowest.

#### 3.5.2. Fed State

In the fed state, the GLP-1 concentrations also significantly differed among diet groups (Figure 2). In this state, groups fed the non-fermentable fibers with no (cellulose) or low viscosity (LV-HPMC) had the numerically highest GLP-1 concentrations, although these groups did not differ from the scFOS and βG groups. There was a significant effect of fermentability (*p =* 0.016) on plasma GLP-1 concentration, such that non-fermentable fibers had a greater GLP-1 concentration than fermentable fibers. Although there was no significant effect of viscosity on plasma GLP-1 concentration in the fed state (*p =* 0.18), there was a trend for the no viscosity fibers to have a greater GLP-1 concentration than the high viscosity fibers (*p =* 0.070).

### 3.6. Proximal Colon GLP-1 Protein Concentration

There were no significant differences among groups in proximal colon GLP-1 tissue concentration (*p =* 0.531), nor were there differences due to fermentability (*p =* 0.986) or viscosity (*p =* 0.433) (data not shown).

### 3.7. Ghrelin

There was a strong overall trend towards a difference in plasma ghrelin concentrations between the fasted and fed states (*p =* 0.057). Fermentability, but not viscosity, significantly influenced the difference in plasma ghrelin between the fasted and fed states (*p =* 0.047).

#### 3.7.1. Fasted State

**Figure 3:**
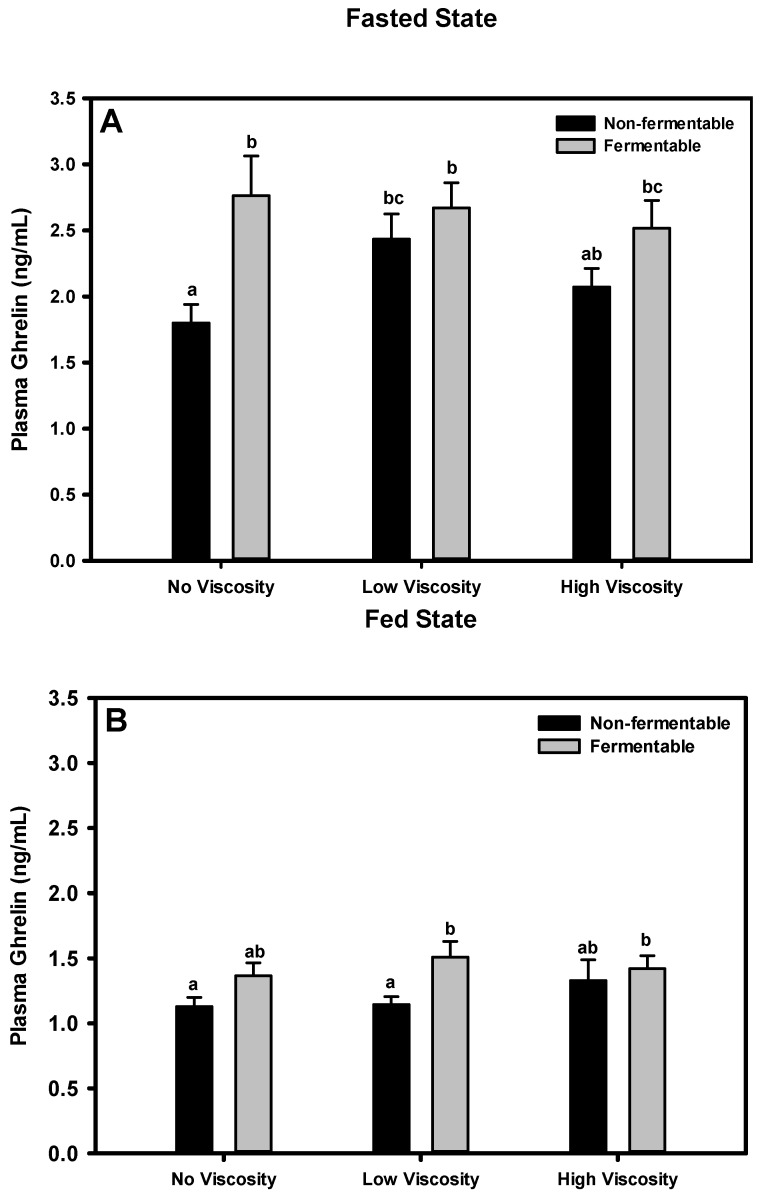
Effect of dietary fiber viscosity and fermentability on plasma ghrelin concentrations in the fasted state (**A**) and fed state (**B**). Values are mean ± SEM, *n* = 10–12 per group. Bars having different letters differ significantly (*p* < 0.05). In the fasted state, there was a significant main effect of fermentability (*p* = 0.001). In the fed state there was also a significant main effect of fermentability (*p* = 0.006). The non-fermentable fibers, in order of increasing viscosity were cellulose, LV-HPMC, and HV-HPMC. The fermentable fibers, in order of increasing viscosity, were scFOS, scFOS + RS, and βG.

In the fasted state, plasma concentrations of ghrelin differed significantly among diet groups (Figure 3). Animals fed the cellulose diet had significantly lower ghrelin concentrations than those fed all other diets except βG. There was an overall significant effect of fermentability (*p =* 0.002) on plasma ghrelin concentration, such that fermentable fibers overall had a greater ghrelin concentration than non-fermentable fibers. The main effect of viscosity was not significant (*p =* 0.350).

#### 3.7.2. Fed State

As expected, the ghrelin concentration overall decreased in the fed state relative to the fasted state (Figure 3). Animals fed the cellulose and LV-HPMC diets had significantly lower ghrelin concentrations than animals on the scFOS + RS and βG diets. The effect of fermentability was statistically significant (*p =* 0.008), such that groups fed the fermentable fibers had a greater ghrelin concentration than the non-fermentable fibers. The effect of viscosity was not statistically significant (*p =* 0.492).

### 3.8. PYY

#### Fasted State

**Figure 4:**
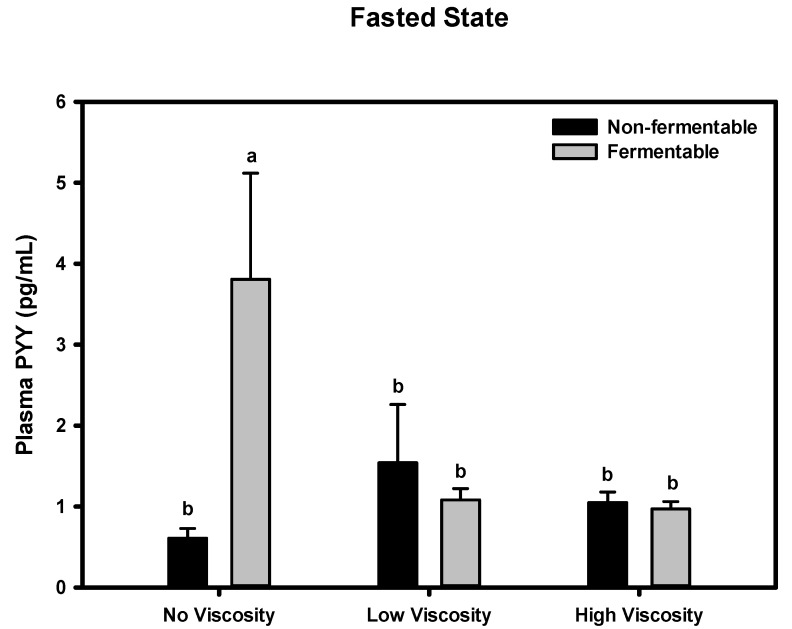
Effect of dietary fiber viscosity and fermentability on plasma PYY concentrations in the fasted state. Values are mean ± SEM, *n =* 10–12 per group. Bars having different letters differ significantly (*p* < 0.05). There was a significant viscosity by fermentability interaction (*p =\* 0.001). The non-fermentable fibers, in order of increasing viscosity were cellulose, LV-HPMC, and HV-HPMC. The fermentable fibers, in order of increasing viscosity, were scFOS, scFOS + RS, and βG.

PYY concentration among diet groups significantly differed (*p* < 0.0001) (Figure 4). Animals fed the scFOS diet had a dramatically greater PYY concentration than all other diets (*p* <0.0001). Although there was a significant viscosity by fermentation interaction for plasma PYY concentration in the fasted state (*p* < 0.001), this was clearly driven by the very high plasma concentration of PYY in the scFOS group.

### 3.9. Leptin

#### 3.9.1. Fasted State

**Figure 5:**
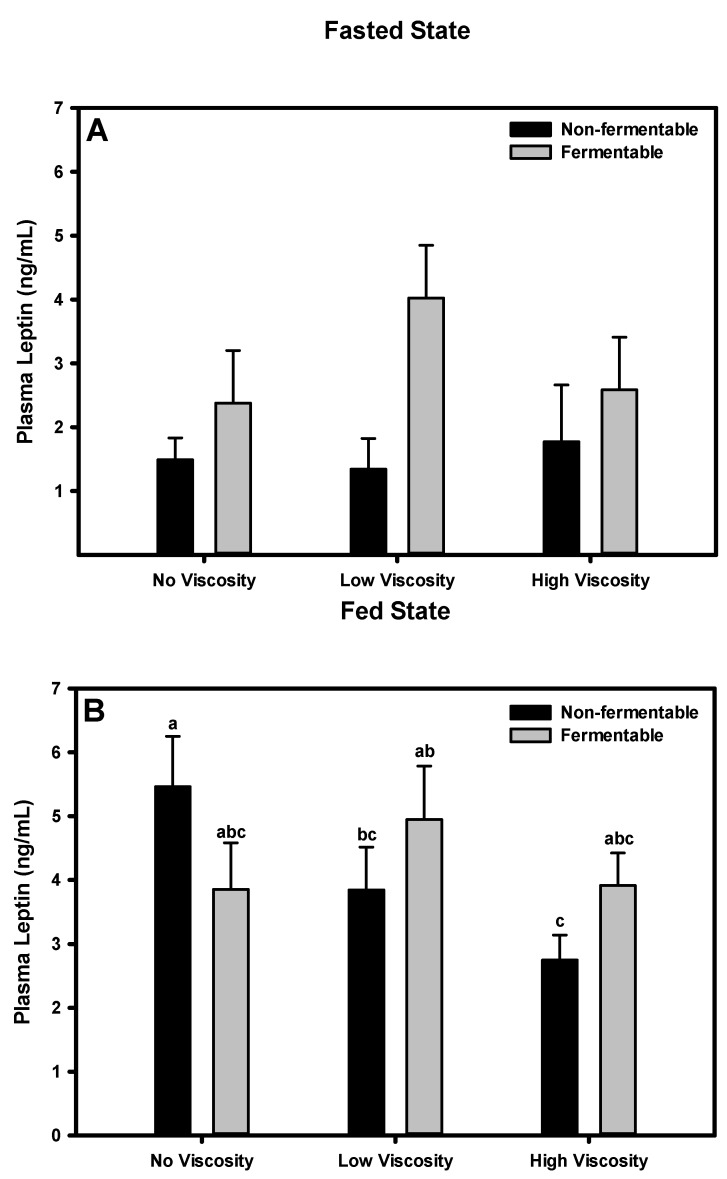
Effect of dietary fiber viscosity and fermentability on plasma leptin concentrations in the fasted state (**A**) and fed state (**B**). Values are mean ± SEM, *n* = 10–12 per group. Bars having different letters differ significantly (*p* < 0.05). In the fasted state, there was a significant main effect of fermentability (*p* = 0.027). In the fed state, there were no significant main effects of fermentation or viscosity. The non-fermentable fibers, in order of increasing viscosity were cellulose, LV-HPMC, and HV-HPMC. The fermentable fibers, in order of increasing viscosity, were scFOS, scFOS + RS, and βG.

Leptin concentrations among diet groups did not differ significantly in the fasted state (*p =* 0.197) (Figure 5). There was an overall significant effect of fermentability on plasma leptin concentration (*p =* 0.027) such that non-fermentable fibers had a lower leptin concentration than fermentable fibers. There was no overall effect of viscosity on leptin concentration (*p =* 0.605).

#### 3.9.2. Fed State

**Figure 6:**
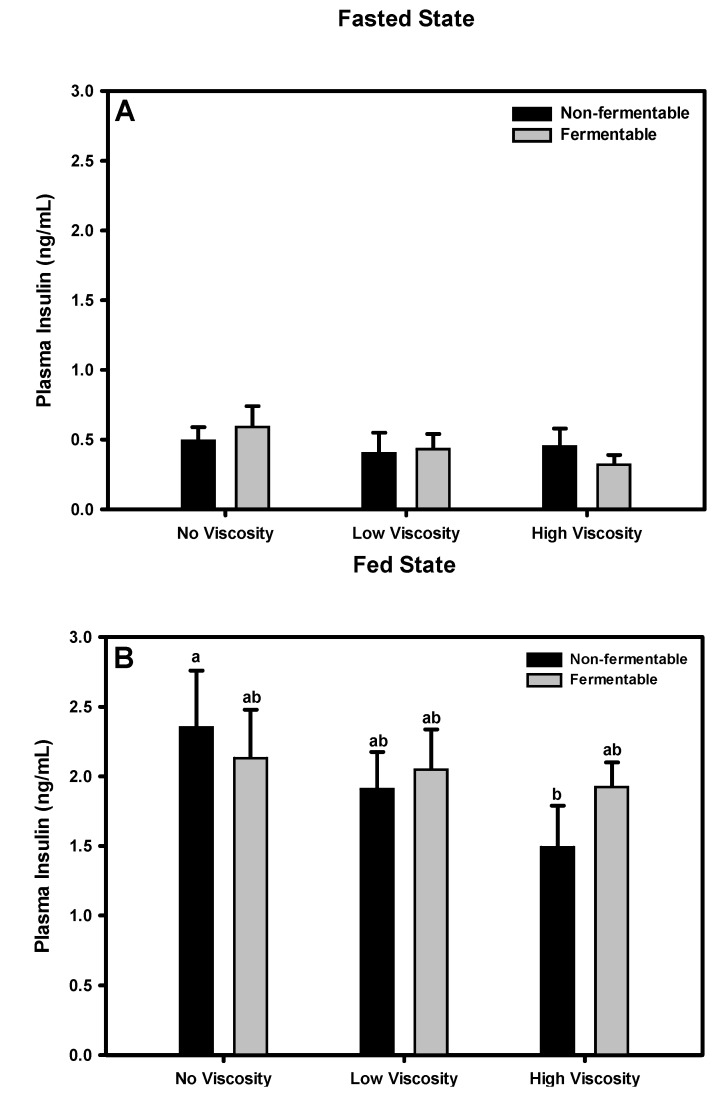
Effect of dietary fiber viscosity and fermentability on plasma insulin concentration in the fasted state (**A**) and fed state (**B**). Values are mean ± SEM, *n* = 10–12 per group. Bars having different letters differ significantly (*p* < 0.05). There were no significant main effects of fermentation or viscosity in either the fasted or fed states. The non-fermentable fibers, in order of increasing viscosity were cellulose, LV-HPMC, and HV-HPMC. The fermentable fibers, in order of increasing viscosity, were scFOS, scFOS + RS, and βG.

The leptin concentration among diet groups differed significantly in the fed state, with the HV-HPMC group having a lower leptin concentration than the cellulose group (Figure 6). There was no significant effect of viscosity or fermentability (*p =* 0.165, *p =* 0.680, respectively); however, there was a trend towards a viscosity × fermentability interaction (*p =* 0.074). That is, the response of leptin to non-fermentable fibers was dependent on the viscosity, such that leptin was lower in non-fermentable fibers with some viscosity.

### 3.10. Insulin

#### 3.10.1. Fasted State

Insulin concentration among diet groups did not significantly differ (*p =* 0.599) in the fasted state. Neither the effects of fermentability (*p =* 0.957) nor viscosity (*p =* 0.322) were statistically significant (Figure 6).

#### 3.10.2. Fed State

In the fed state, plasma insulin concentration in the HV-HPMC group was significantly less than in the cellulose group. However, there were no significant main effects of fermentability (*p =* 0.625) or viscosity (*p =* 0.221) (Figure 6).

### 3.11. Correlations between Hormones

Table 3 shows correlations between plasma hormone concentrations. Fed insulin levels were positively correlated with fed leptin levels. Fasting and fed insulin levels were inversely correlated with fasting ghrelin levels. Fasting insulin was inversely correlated with fed GLP-1 whereas fed insulin levels inversely correlated with GLP-1 tissue amount (Table 3). Fasting GLP-1 levels were positively correlated with fasting PYY.

**Table 3:** Correlations between plasma hormone concentrations. Data presented as correlation coefficients and corresponding *p*-values. Abbreviation: NS, not statistically significant.

	Fasting Leptin	Fed Leptin	Fasting Ghrelin	Fed Ghrelin	Fasting PYY	Fed GLP-1	Tissue GLP-1
Fasting Insulin	NS	0.24 *p =* 0.059	−0.29 *p =* 0.021	NS	NS	−0.25 *p =* 0.048	NS
Fed Insulin	NS	0.52 *p* <0.0001	−0.26 *p =* 0.039	NS	NS	NS	−0.26 *p =* 0.040
Fasting GLP-1	NS	NS	NS	NS	0.37 *p =* 0.003	NS	−0.29 *p =* 0.021

### 3.12. Stepwise Regression of Average Food Intake with Plasma Hormones

In order to understand how the plasma concentration of the various hormones, measured in different physiological states, may have influenced food intake, a stepwise regression of hormone concentrations against average food intake was conducted (Table 4). All plasma hormone concentrations, from both fasted and fed states, were used in the model. Fed state leptin, fasted state ghrelin, and fasted state PYY were all statistically significant variables in the regression model and together, with a cumulative *R*^2 ^= 0.496, indicating that these three variables combined explained approximately 50% of the variability in food intake.

**Table 4:** Results of stepwise regression of average food intake with plasma hormone concentrations.

Hormone-state	Partial *R*^2^	Cumulative *R*^2^	*P* Value for Each Parameter
Leptin-fed	0.181	0.181	0.0008
Ghrelin-fasted	0.201	0.381	0.0001
PYY-fasted	0.114	0.496	0.0008

## 4. Discussion

A number of previous studies have examined the influence of different sources of dietary fiber on satiety- and adiposity-related hormones. However, to date, the influence of fiber viscosity and fermentability, the two most physiologically important characteristics of dietary fiber, on satiety- and adiposity-related hormones has not been systematically examined. This study was designed to investigate how these two fiber characteristics influence the plasma concentration of satiety- and adiposity-related hormones. Further, plasma concentrations of all hormones except PYY were determined in both the fasted and fed states in order to understand how the presence or absence of dietary fiber within the intestinal tract may influence plasma hormone concentrations. Our results suggest that the relationship between satiety- and adiposity-related hormones and dietary fiber characteristics is complex. For example, both non-fermentable and fermentable fibers may enhance satiation or satiety, depending on the viscosity of the fiber. Further, whether the animal is in the fasted or fed state also influences the relationship.

There was a significant interaction between fiber viscosity and fermentability in final body weight, with a trend toward lower body weight with increasing viscosity of non-fermentable fibers, but an opposing trend towards a greater body weight with increasing viscosity of fermentable fibers. Indeed, animals consuming HV-HPMC, the most viscous fiber, but one that is non-fermentable, had the lowest final body weight. Although average daily food intake did not differ among the groups, there was a strong and statistically significant correlation between the group means of final body weight and average daily food intake. The HV-HPMC group had the lowest daily food intake, a finding that would be consistent with greater satiety in this group. Viscous fibers, however, may increase satiety by mechanisms other than changes in plasma satiety-related hormone concentration, including increasing gastric distention, delayed gastric emptying, or activation of the ileal brake. Of these, only gastric emptying has been examined in regards to fiber viscosity, and the results are inconsistent, with some studies supporting delayed gastric emptying with greater viscosity [2,3,4] and others not [5,6]. Plasma ghrelin concentrations increase postprandially, but a slower gastric emptying rate will dampen this increase [7]. Thus, the lack of an effect of fiber viscosity on the postprandial ghrelin response (Figure 3) provides indirect evidence for a lack of an effect of fiber viscosity on the gastric emptying rate. The HV-HPMC group also had the lightest epididymal fat pad weight, with the cellulose group having the heaviest. This is consistent with the findings of others [8,9], who found rats fed a high viscosity HPMC to also have lighter fat pad weights compared to a cellulose-fed control. The scFOS group also had a lower final body weight, compared to the cellulose group, and equivalent to that of the HV-HPMC group. Others have reported lower weight gain with fructooligosaccharide or inulin feeding compared to control groups [1,10], although in those studies energy intake was lowered with consumption of these fructans. In the present study, the lower final body weight of the scFOS group occurred with energy intake statistically equivalent to the cellulose group. However, the strong correlation between final body weights and food intake suggests that small differences in food intake between the scFOS and cellulose groups, over time, led to the observed differences in final body weight. The FOS + RS group, whose diet contained 1.24% resistant starch, did not have a lower body weight or fat pad weight than the scFOS group. Resistant starch consumption at approximately 20% of the diet has been associated with both a reduced weight gain and greatly reduced adiposity [11]. It has been suggested that this reduction in adiposity may involve a decrease in metabolizable energy intake [12]. However, given that the net energy provided by highly fermentable fibers is much greater than that of non-fermentable fibers [13], this suggestion seems unlikely. Regardless, it appears that much higher dietary resistant starch concentrations than that present in the FOS+RS diet must be fed to see reductions in adiposity, as only diets containing ≥8% resistant starch reduced adiposity in rats [12]. Regardless, the mechanism by which HV-HPMC and scFOS reduce final body weight cannot be determined from this study, but would seem to warrant further investigation.

As expected, animals consuming the fermentable fibers had significantly heavier cecum and cecal contents weights but, surprisingly, lighter proximal colon weights than animals consuming non-fermentable fibers. Fermentable fibers are well known to induce enlargement of the cecum [14,15,16], as least in part due to the trophic effects of short chain fatty acids, products of fermentation [17,18]. The greater cecum contents weight is likely due to the increased bacterial mass with fermentation and the greater amount of water that would accompany it. However, it is not obvious why fermentation would tend to lead to lighter proximal colon weights. Perhaps the greater bulk of material passing into the colon from the cecum with non-fermentable fibers leads to a greater tissue mass. In contrast, there was no significant effect of fiber viscosity on either cecal contents weight or proximal colon weight, but there was a significant interaction for cecum weight, driven primarily by the low cecum weight of the scFOS + RS group relative to the two other fermentable fiber groups. Thus, a small amount of resistant starch appeared to counter the trophic effect of the scFOS on cecum weight.

Viscosity, overall, had no significant influence on plasma concentrations of GLP-1, ghrelin, or PYY in either the fasted or fed states. However, there were individual group differences that allow a degree of comparison to other studies. Vitaglione *et al.* [19] fed subjects a meal of either a whole wheat bread or a bread containing β-glucans. At two hours after bread consumption plasma ghrelin concentration was significantly lower in the group consuming the β-glucan enriched bread. In contrast, in the present study at two hours postprandial, the β-glucan fed group had a significantly greater plasma ghrelin concentration that the cellulose control group. Karhunen *et al.* [20] fed subjects a low fiber meal or a meal containing psyllium, a gel-like viscous fiber with limited fermentability. At two hours postprandial plasma GLP-1 concentrations were similar between the two meals, whereas plasma ghrelin concentrations differed somewhat between the two meals, with ghrelin actually higher than baseline (fasting) values with the psyllium meal, whereas the low fiber meal resulted in ghrelin concentrations similar to the baseline values. In the present study, in all groups, plasma GLP-1 and ghrelin concentrations declined from the fasted state to the fed state. The magnitude of this decline was similar for the cellulose and HV-HPMC groups, the two groups most comparable to the low fiber and psyllium meals, respectively. These inconsistencies in the effect of viscous fiber on plasma ghrelin could represent a species difference, a difference between a single meal *versus* chronic feeding of a viscous fiber, or both. Finally, it has been suggested that a viscous product may slow the gastric emptying rate and thereby delay the decrease in ghrelin levels usually apparent postprandially, as nutrients would be released from the stomach into the small intestine at a slower rate [7,21]. However, our finding that fiber viscosity did not alter the concentration of ghrelin postprandially is not consistent with this possibility. 

In contrast to the lack of an overall effect of viscosity on satiety-related hormones, fermentability had a significant effect on plasma GLP-1 concentrations in the fed state and on plasma ghrelin concentrations in both the fasted and fed states. Overall, fermentable fibers had lower plasma GLP-1 concentrations than non-fermentable fibers in the fed state, and in the fasted state, which showed a viscosity by fermentability interaction, the low and high viscosity fermentable fiber groups had lower plasma GLP-1. In the case of fibers with no viscosity, however, the fermentable fiber scFOS resulted in greater plasma GLP-1 concentrations than non-fermentable cellulose. This is consistent with several studies reporting that fructans such as fructooligosaccharides or inulin increase GLP-1 concentrations in the portal vein [10,22]. Resistant starch, another highly fermentable fiber, has also been shown to increase plasma GLP-1 [11,23]. However, in the present study, the group fed the fiber source containing >80% scFOS, with the remainder resistant starch, that is the scFOS + RS group, had a lower concentration of plasma GLP-1 compared to the cellulose-fed group in both the fasted and fed states. Other studies in which fructans compose only part of the dietary fiber are inconsistent, with one study showing an increased GLP-1 concentration [24] and another no change [25], relative to the low fiber control. Given that infusion of short chain fatty acids into a vascularly perfused rat colon did not induce a significant release of GLP-1 [26], the greater plasma GLP-1 in the scFOS group in the fasted state may be due to factors other than fermentation products. Thus, it appears that the influence of fermentable fibers on plasma GLP-1 warrants further investigation.

There were no significant overall effects of fermentability or viscosity, nor any significant differences among diet groups, for proximal colon GLP-1 concentration. Others have found highly fermentable fibers to increase proglucagon expression in dogs, rats, and mice compared to cellulose [1,23,24,27] or methylcellulose fiber control groups [11]. Since proximal colon GLP-1 concentration has been found to correlate with proglucagon expression [27], it would be expected that in these studies proximal colon GLP-1 tissue concentration would have increased as well.With this understanding, our findings are not in agreement with these studies. Although in two of these studies the fermentable fiber was fed in combination with a high fat diet [1,27], and fat appears to be the most important nutrient causing release of GLP-1 [28], it is not clear this can explain the inconsistency. Possibly more relevant is that we obtained the proximal colon in fed state, whereas other studies used colon tissue obtained in the fasted state. The greater plasma GLP-1 concentration in the fasted state with the fermentable fiber with no viscosity (scFOS) provides some support for this explanation.

Ghrelin, as an orexigenic hormone, increases in concentration during fasting (signaling hunger) and decreases in the fed state (causing meal termination). As expected, there was an overall decrease in ghrelin concentration in the fed state compared to the fasted state. However, in both the fasted and fed states, fermentable fibers had significantly greater ghrelin concentrations compared to the non-fermentable fibers, suggesting that fermentable fibers may be associated with less satiety than non-fermentable fibers. Our findings are not in agreement with those of others. Cani *et al.* [10] found that, in rats, feeding fructans resulted in lower concentrations of plasma ghrelin in the fasted state compared to a cellulose-fed control group. In dogs fed a fermentable fiber mixture (sugar beet pulp and inulin) plasma ghrelin, both in the fasted state or after a meal, did not differ from that of animals fed cellulose [25]. Thus, the influence of fermentable fibers on plasma ghrelin appears uncertain.

Plasma PYY was measured only in the fasted state. The significant main effect of fermentability on PYY concentrations was driven by the dramatically greater PYY concentration in the scFOS group relative to all other groups. In the vascularly perfused rat colon, butyrate, and to a lesser extent propionate, but not acetate, was shown to enhance PYY release into the portal effluent [29]. As fructans such as fructooligosaccharides and inulin increase production of butyrate [30,31], the increase in plasma PYY with scFOS feeding may represent a response that is specific to fructans, and not necessarily to other fermentable fibers. 

Leptin and insulin may be more appropriately categorized as adiposity signals as opposed to satiation or satiety signals, as both are secreted in proportion to total body fat [32,33]. However, leptin and insulin indirectly affect satiety by influencing sensitivity to satiety signals. For example, the satiety effect of GLP-1 is lessened in the absence of leptin signaling, suggesting GLP-1 is dependent on leptin for inducing satiety [34]. Similarly, PYY levels are influenced by caloric load and food composition [35] and its effect may depend on energy balance and leptin signaling [36]. In the present study, a lower plasma leptin concentration in the fasted state in non-fermentable fibers suggests these fibers may be associated with lower adiposity and greater satiety than fermentable fibers. In the fed state there was no overall effect of either viscosity or fermentability on plasma leptin, nor an interaction. However, both the LV-HPMC and HV-HPMC groups had lower plasma leptin concentrations than the cellulose group, in agreement with previous studies [9], and there was a strong trend for lower plasma leptin in the scFOS group. Thus, in non-fermentable viscous fibers, leptin may play a role in increasing satiety, thereby decreasing food intake and leading to a lower body weight gain.

Although neither viscosity nor fermentability had an overall effect on plasma insulin, the HV-HPMC group displayed significantly lower insulin concentration than the cellulose group in the fed state, as has been reported by others [9,37]. The postprandial reduction in insulin with viscous fiber feeding has generally been ascribed to either a delay in gastric emptying or a slowed diffusion of glucose within the small intestine [4,38]. The influence of a reduced postprandial insulin response on satiety is uncertain, however, as it is elevated plasma insulin concentrations have been shown to suppress food intake [39].

## 5. Conclusions

We found that both non-fermentable and fermentable fibers may influence body weight and satiety- and adiposity-related hormone concentrations. The strong and statistically significant correlation between final body weight and daily food intake is consistent with differences in final body weight being mediated by differences in satiety. Paradoxically, the two groups with the lowest body weight were fed a non-viscous fermentable fiber (scFOS) and a highly viscous non-fermentable fiber (HV-HPMC). Overall, however, when fermentability was found to have a significant effect on satiety hormone concentration, the trend was for changes in the direction of greater satiety in groups fed non-fermentable fibers. Thus, non-fermentable dietary fibers warrant consideration for incorporation into foods to promote satiation and satiety and thereby provide an important tool for improving body weight management.
